# Attentional disengagements in educational contexts: a diary investigation of everyday mind-wandering and distraction

**DOI:** 10.1186/s41235-017-0070-7

**Published:** 2017-08-23

**Authors:** Nash Unsworth, Brittany D. McMillan

**Affiliations:** Department of Psychology, University of Oregon, Eugene, OR 97403 USA

**Keywords:** Mind-wandering, External distraction, Cognitive abilities, Motivation, Interest

## Abstract

The present study examined everyday attentional disengagements in educational contexts. Undergraduate students completed various cognitive ability measures in the laboratory and recorded everyday mind-wandering and distraction in a diary over the course of a week. Participants reported mind-wandering and being distracted both in class and while studying and there were a number of different subtypes of attentional disengagements. Individual differences in cognitive abilities were related to some, but not all, everyday attentional disengagements and motivation and interest in classes were related to specific subtypes of disengagements. Finally, academic performance was related to fluid intelligence and motivation, but not to everyday disengagements. These results provide importance evidence on the different types of attentional disengagements that are prevalent in undergraduate students and for whom disengagements are most likely.

## Significance

Mind-wandering and distraction are two potent forms of attentional disengagements that plague students both in class and while studying. In the current study undergraduate students performed a number of cognitive ability measures in the laboratory and logged their attentional disengagements in a diary over the course of a week. The results suggested that both mind-wandering and distraction occur in class and while studying. Furthermore, various subtypes of attentional disengagements were identified suggesting that some of the most frequent disengagements were being distracted by surrounding conversations and other information, self-distraction with technology, and mind-wandering because of boredom and disinterest in the course material. Individual differences in cognitive abilities, motivation, and interest predicted various disengagements. The current study provides key evidence that attentional disengagements are a common hindrance to undergraduate students and there are important individual differences in the susceptibility to everyday mind-wandering and external distraction.

## Background

Our ability to focus and sustain attention is a hallmark of a highly functioning attentional system. This attentional system allows us to perform both highly important as well as mundane tasks. Despite the efficiency of such a system, sometimes we are not fully engaged in the task at hand, but rather are captured by potent internal or external distraction. For example, have you ever been distracted by people talking while you were trying to grade exams? Have you ever found yourself zoning out during a meeting because you were daydreaming about your weekend plans? Most of us will likely answer in the affirmative to these questions. These attentional disengagements reflect temporary shifts of attention away from the task at hand to either external stimuli (distractions) or to internal thoughts and ruminations (mind-wandering/daydreaming) that can result in failures to perform an intended action. Although there may be some benefits to these lapses of attention (e.g., attentional capture towards a threat stimulus, problem solving an unrelated task), for the most part these attention failures are seen as unwanted breakdowns of our attentional system. As such, attention failures have been linked to both minor and major accidents (Broadbent, Cooper, FitzGerald, & Parkes, 1982; Reason & Mycielska, 1982), as well as to professional (Reason, 1990), and educational difficulties (Brown, 1927; Lindquist & McLean, 2011). Although a great deal of work has been done examining how our attentional system operates using basic laboratory paradigms, considerably less work has been done examining the attentional system in more ecologically valid contexts. Given the importance of our attentional system in a diverse array of situations, it is clear that more research is needed to systematically examine when, and for whom, the attentional system is most likely to falter in real-world situations.

In the current study we examined attentional disengagements in educational contexts (in class and while studying) in a sample of undergraduate students. By attentional disengagements we mean those situations in which attention is disengaged from the primary task (listening to a lecture and taking notes, studying for an upcoming exam, etc.) and is instead allocated to irrelevant internal or external sources (see also Cheyne, Solman, Carriere, & Smilek, 2009). These shifts in attention may be voluntary in which attention is intentionally disengaged from the primary task and instead allocated to other internal (thinking about what you have to do later; e.g., Seli, Risko, & Smilek, 2016) or external (checking your email on your phone) sources of information. These voluntary shifts are likely linked to motivation and interest such that when one is less motivated and/or less interested in the primary task, attention is allocated to off-task activities (Kanfer & Ackerman, 1989). These shifts may also be involuntary in which attention is disengaged from the primary task due to potent internal (current concerns; Klinger, 2013) or external (loud noises) distractions that capture attention. These involuntary shifts of attention may be linked to overall alertness and arousal levels (Kahneman, 1973) such that when underaroused and bored, less attention is devoted to remaining engaged in the current task, and thus more easily captured by potent distractors (Lenartowicz, Simpson, & Cohen, 2013). Thus, in the current paper we refer to attentional disengagements as situations where students’ attention is disengaged from what they are supposed to be doing (taking notes, studying) to other internal and external sources of information. In prior research we (Unsworth, McMillan, Brewer, & Spillers, 2012) and others referred to these as attentional failures given that attention was supposed to be allocated to a given task, but was instead allocated to irrelevant information resulting in a failure or lapse of attention. However, given that sometimes attention can be voluntarily allocated to things other than the primary task (checking Facebook because I find the lecture boring), we refer to the broad class of situations where attention is allocated to other sources as disengagements rather than as failures (which can be considered as a subclass of overall disengagements).

### Mind-wandering and external distraction in educational contexts

It has long been recognized that attentional disengagements in educational contexts (i.e., while studying or in class) are a major hindrance to students’ ability to learn and retain information. For example, early reports suggested that students spend 10–40% of class time thinking about topics unrelated to the class (Schoen, 1970). Furthermore, recent research has suggested that students begin to experience disengagements (mind-wandering or external distraction) within the first 5 min of class with increasing frequency of disengagements as the lecture progresses (Bunce, Flens, & Neiles, 2010). Additionally, recent research suggests that roughly 76% of college students’ disengagements occur either in class or while studying (Unsworth, McMillan, et al., 2012). Clearly, understanding the nature of the these attentional disengagements and for whom, and under what circumstances, they are most frequent is important in order to not only understand how the attentional system operates in the real world but to also devise better interventions to keep students focused in class and while studying to ensure better learning and retention of material.

Prior research has suggested that students repeatedly report that much of the time during lectures or while studying is spent mind-wandering or daydreaming about topics unrelated to the current material (Brown, 1927; Szpunar, Moulton, & Schacter, 2013). Mind-wandering refers to the phenomena in which attention is shifted away from external information to internal thoughts and feelings that are unrelated to the current task at hand (McVay & Kane, 2010; Smallwood & Schooler, 2006). A number of laboratory techniques have been developed to examine mind-wandering including thought-probe techniques in which periodically throughout a task participants are probed as to their current state (on-task or off-task) and this is examined as a function of various experimental manipulations and individual differences correlates (Antrobus, 1968; see Smallwood & Schooler, 2006, 2015 for reviews). These thought-probe techniques have also been shown to predict mind-wandering in everyday life (e.g., Kane et al., 2007; Kane et al., in press) and mind-wandering in the laboratory and in everyday life tend to correlate (McVay, Kane, & Kwapil, 2009). Typically rates of mind-wandering correlate with task performance such that performance is lower when participants report mind-wandering on the preceding trial compared to when participants report that they are focused on the current task (McVay & Kane, 2010; Smallwood & Schooler, 2006). Furthermore, research has suggested that mind-wandering varies as a function of task variables such as time on task, task complexity, and task difficulty (McVay & Kane, 2010; Smallwood & Schooler, 2006).

A number of studies suggest that roughly 10–50% of the time during lectures is spent mind-wandering or in an inattentive state (Cameron & Giuntoli, 1972; Geerligs, 1995; Lindquist & McLean, 2011; Wammes, Boucher, Seli, Cheyne, & Smilek, 2016) and this increases as the lecture progresses (Lindquist & McLean, 2011; Risko, Anderson, Sarwal, Engelhardt, & Kingstone, 2012; Szpunar, Khan, & Schacter, 2013; but see Wammes, Boucher, et al., 2016). Furthermore, Siegel, Siegel, Capretta, Jones, and Berkovitz (1963) demonstrated that reports of mind-wandering during a classroom lecture predicted later comprehension scores of the lecture material (see also Lindquist & McLean, 2011; Wammes, Seli, Boucher, Cheyne, & Smilek, 2016). Likewise, Risko et al. (2012) had laboratory participants watch a recorded lecture and found that reports of mind-wandering were negatively related with comprehension scores (see also Hollis & Was, 2016; Risko, Buchanan, Medimorec, & Kingstone, 2013; Seli, Wammes, Risko, & Smilek, 2016; Szpunar, Khan, et al., 2013). Similar relations are also found when laboratory participants are instructed to study educational texts and then take a test on the material. Specifically, higher rates of mind-wandering while studying a political science text were related with lower scores on a subsequent test of the material (Robison & Unsworth, 2015; Unsworth & McMillan, 2013). A recent diary study where college students were required to report on their attentional failures over the course of a week, found that two of the most common attentional failures were mind-wandering in class (15% of total failures) and mind-wandering while studying (5% of total failures; Unsworth, Brewer, et al., 2012; Unsworth, McMillan, et al., 2012). Thus, not only do students routinely report mind-wandering during lectures and while studying, but higher rates of mind-wandering are negatively related with scores on later tests of learning and comprehension.

Students also report being distracted by information in the external environment a significant portion of the time. For example, in the same diary study discussed above it was found that 31% of the reported failures were due to distractions either while studying (22%) or while in class (9%). Like mind-wandering, external distraction can also impede learning and retention. For example, Shelton, Elliott, Eaves, and Exner (2009) found that hearing a ringing cell phone during a lecture resulted in lower scores for material presented at the same time as the ringing cell phone compared to material presented before the ringing cell phone. External distraction, in particular auditory distraction, is frequently reported as being disruptive in both work and school environments (Beaman, 2005). In such situations attention is shifted from the current task to irrelevant (and potentially annoying) information in the external environment. Thus, like mind-wandering external distraction represents a disengagement of attention where attention is captured by task-unrelated information. External distractors can take on multiple forms including extraneous noises (such as talking, laughing, coughing, ringing cell phones, or other ambient noises) extraneous sights (such as lighting conditions, people playing games on their computers, or others moving around), temperature fluctuations (too hot or too cold), odors, among many others. In fact, in a recent study Tesch, Coelho, and Drozdenko (2011) found that students talking with others in class, ringing cell phones, and students coughing were some of the most reported external distractors experienced by college students during lectures. External distractors such as ringing cell phones and irrelevant speech have consistently been shown to reduce performance on comprehension tests for material learned in the presence of the distractors (e.g., Banbury & Berry, 1998; Shelton et al., 2009; Sörqvist et al. 2010; Zeamer & Fox Tree, 2013). For example, Zeamer and Fox Tree (2013) examined how different types of auditory distraction would impact comprehension of lecture material while participants watched recorded lectures. Zeamer and Fox Tree found that irrelevant speech, laughter, and unusual noises (i.e., construction noise, people coughing and sneezing) all negatively impacted comprehension of the lecture material. When asked about the nature of the noise, participants routinely mentioned being distracted by the various types of noises. Thus, like mind-wandering environmental factors such as external distractors can lead to disengagements of attention and changes in subjective attentional state (Damrad-Frye & Laird, 1989). In addition to types of distractors, other factors are important including the overall intensity or loudness of the extraneous noise (Damrad-Frye & Laird, 1989; Szalma & Hancock, 2011). With moderate levels of distraction few participants actually report being distracted, rather they report being bored an uninterested with the material they are attempting to comprehended (Damrad-Frye & Laird, 1989). With higher levels of distraction, however, nearly all participants report some level of distraction due to the extraneous noise (Damrad-Frye & Laird, 1989).

Another major distractor in educational settings is the use of technology (computers and smartphones). Fried (2008) reported that 64% of participants indicated that other students using laptops was a distractor and Jacobsen and Forste (2011) reported that 62% of participants indicated using electronic media for nonacademic purposes while in class and while studying. Furthermore, Fried (2008) demonstrated that computer use was negatively related to overall learning. Similarly, Sana, Weston, and Cepeda (2013) showed that computer use resulted in lower performance for the user and for peers’ nearby computers. Likewise, monitoring eye movements, Phillips, Ralph, Carriere, and Smilek (2016) found that participants were more distracted if the person in front of them was watching a video on their laptop compared to simply reading an article on the laptop or having the laptop closed. Additional research has shown that students who attempt to multi-task by trying to attend to lectures and attend to non-lecture-related computer content at the same time have poorer learning and retention than students who do not use computers (Jacobsen & Forste, 2011; Ravizza, Hambrick, & Fenn, 2014; Risko et al., 2013; Wood et al., 2012). Non-lecture-related content, such as using Facebook during lectures, is negatively related to student engagement (Junco, 2012). Likewise, cell phone use during class is negatively related to subsequent grades (Bjornsen & Archer, 2015) and thoughts about technology (such as thinking about texting or thinking about using Facebook) are negatively related to academic performance (Hollis & Was, 2016). Furthermore, recent research has found that laptop use was negatively related to overall interest in the course and final exam scores (Ravizza, Uitvlugt, & Fenn, 2017). Likewise, propensity to engage in nonacademic technological distractions (checking email, checking Facebook, playing music, etc.) during study has been found to be negatively associated with class motivation and self-efficacy (Calderwood, Ackerman, & Conklin, 2014). In terms of educational contexts these results suggest that attentional disengagements are likely in conditions where there are multiple different types of external distractors. Given that most classroom and study environments include a variety of external distractors, it is quite likely that students not only mind-wander frequently during lectures and while studying, but that they are also frequently distracted by information in the environment (Tesch et al., 2011; Unsworth, Brewer, et al., 2012; Unsworth, McMillan, et al., 2012). Collectively, prior research demonstrates the pervasiveness of attentional disengagements in educational contexts and suggests that both mind-wandering and external distraction can negatively impact learning and retention of course material.

### Influence of person factors on mind-wandering and external distraction

Person factors, such as individual differences in cognitive abilities, motivation, and interest, are also likely important for determining who is most likely to experience disengagements of attention. For example, it has been recognized that individuals vary in their capacity for attention control with lower ability individuals experiencing more lapses of attention than higher ability individuals. Recent research on working memory capacity (WMC) has suggested that individual differences in this construct are an important indicator of individual differences in attention control that are needed in a variety of situations where task goals must be maintained in the presence of internal and external distraction (Engle & Kane, 2004; Unsworth & Engle, 2007). A number of studies have demonstrated strong relations between WMC, attention control, and intelligence (Kane et al., 2016; McVay & Kane, 2012; Unsworth & McMillan, 2014; Unsworth, Redick, Lakey, & Young, 2010; Unsworth, Fukuda, Awh, & Vogel, 2014). Furthermore, recent research suggests that the relation between WMC and higher-order cognition (such as comprehension) is due, in part, to variation in mind-wandering (McVay & Kane, 2012; Unsworth & McMillan, 2013). For example, Unsworth and McMillan (2013) found that WMC positively predicted reading comprehension scores for material from an introductory political science text. Importantly, self-reports of mind-wandering while reading the text largely mediated this effect. That, is low-WMC individuals reported more mind-wandering than high-WMC individuals and these differences in mind-wandering largely accounted for differences in subsequent test scores. Given the tight relation between WMC and attention control, it is perhaps not surprising that individual differences in WMC predict individual differences in lapses of attention in both laboratory and daily life situations. For example, prior research has shown that low-WMC individuals report both more mind-wandering during challenging everyday tasks (Kane et al., 2007; Kane et al., in press; Marcusson-Clavertz, Cardena, & Terhune, 2016; Unsworth, Brewer, et al., 2012; Unsworth, McMillan, et al., 2012) and more external distraction during everyday tasks (Unsworth, Brewer, et al., 2012; Unsworth, McMillan, et al., 2012). Furthermore, although both mind-wandering and external distraction can be considered as attentional disengagements, recent research suggests that these two constructs are positively related but are not isomorphic (Robison & Unsworth, 2015; Stawarczyk, Majerus, Catale, & D’Argembeau, 2011; Unsworth, Brewer, et al., 2012; Unsworth, McMillan, et al., 2012; Unsworth & McMillan, 2014). Indeed, in a recent study (Unsworth & McMillan, 2014) participants performed multiple measures of WMC and multiple attention control tasks (antisaccade, Stroop, flankers, etc.) and were probed periodically to determine if they were on-task, mind-wandering, or distracted by information in the environment. It was found that rates of mind-wandering and external distraction were positively correlated (see also Unsworth, Brewer, et al., 2012; Unsworth, McMillan, et al., 2012) but the correlation did not approach 1.0. Furthermore, both mind-wandering and external distraction were negatively correlated with WMC, attention control, and fluid intelligence. More recently, Robison and Unsworth (2015) had participants read excerpts from a political science textbook either in silence or in the presence of background noise (a recording from a busy bar/restaurant). Replicating prior research, WMC was related to self-reports of mind-wandering in the silent condition. However, in the noise condition WMC was related to self-reports of external distraction, but not mind-wandering. Furthermore, mind-wandering was negatively related to comprehension scores in both conditions, but external distraction was only related to comprehension in the noise condition. These results suggest that there are important individual differences in the susceptibility to mind-wandering and external distraction (which are somewhat dependent on the presence or absence of distraction in the environment) and that these individual differences are related to individual differences in other cognitive abilities. Thus, person factors in terms of variation in cognitive abilities influence the likelihood of mind-wandering and external distraction and their relation to learning and comprehension of material.

Other important person factors include noncognitive factors such as interest and motivation. For example, a large body of research has consistently demonstrated that interest in the text that one is learning has a strong impact on overall comprehension scores (e.g., Baldwin, Peleg-Bruckner, & McClintock, 1985; Hidi, 2001; Schiefele & Krapp, 1996; Tobias, 1994). The more interested one is in the topic at hand, the more likely one is to deeply learn the material leading to overall better comprehension scores. It has also been suggested that topic interest and attentional allocation are related such that the more interested one is in the current topic the better their attention is focused on the material leading to overall deeper processing of the text (Hidi, 2001). Additionally, topic interest has been linked with motivational factors suggesting that the more interested one is in the topic of the text, the more motivated they are to learn and perform well on subsequent comprehension tests (e.g., Hidi & Harackiewicz, 2000). Indeed, several studies have specifically examined the influence of motivation on learning and comprehension and have consistently found that motivation (both intrinsic and extrinsic) is a strong predictor of test scores (Anmarkrud & Braten, 2009; Guthrie et al., 2007; Guthrie, Wigfield, Metsala, & Cox, 1999) and individual differences in motivation are related to academic performance (Pintrich, 1999; Schiefele, Krapp, & Winteler, 1992; Steinmayr & Spinath, 2009). Like WMC, the influence of interest and motivation on test scores is likely partially due to their influence on mind-wandering. For example, Giambra and Grodsky (1989) found a negative relation between interest and mind-wandering while reading such that participants reported more mind-wandering for texts they found to be uninteresting compared to texts they found to be interesting. Furthermore, motivational factors also seem to play a role in mind-wandering such that when motivation is high to perform well (based on financial incentives) mind-wandering is reduced (Antrobus, Singer, & Greenberg, 1966; Mrazek et al., 2012). Thus, cognitive and noncognitive factors are important when examining the relation between disengagement of attention and learning (Kanfer & Ackerman, 1989). Recent studies support these notions (Hollis & Was, 2016; Robison & Unsworth, 2015; Unsworth & McMillan, 2013). For example, Unsworth and McMillan (2013) found that WMC significantly predicted mind-wandering and mind-wandering partially mediated the relation between WMC and reading comprehension. Additionally, interest and motivation predicted mind-wandering and mind-wandering fully mediated the relations between interest and motivation with reading comprehension. Importantly, WMC was not related to either interest or motivation (see also Robison & Unsworth, 2015). Similarly, Seli, Wammes, et al. (2016) found that individual differences in motivation predicted mind-wandering while watching an online lecture, and mind-wandering mediated the relation between motivation and retention. Likewise, Hollis and Was (2016) found that individual differences in interest levels predicted mind-wandering rates, and mind-wandering mediated the relation between interest and subsequent performance. These results strongly suggest that person factors, such as cognitive abilities, interest, and motivation, are related to the likelihood of experiencing attentional disengagements (in this case mind-wandering), and these disengagements influence learning and comprehension of course materials.

### Present study

The goal of the present study was to replicate and extend prior research which has examined attentional disengagements in educational contexts (Unsworth, McMillan, et al., 2012). In particular, we were interested in examining mind-wandering and external distraction both in class and while studying. Although much prior work has specifically examined mind-wandering in both real classes (e.g., Cameron & Giuntoli, 1972; Geerligs, 1995; Lindquist & McLean, 2011; Wammes, Boucher, et al., 2016) and simulated lectures (e.g., Hollis & Was, 2016; Risko et al., 2012, 2013; Seli, Wammes, et al., 2016; Szpunar, Khan, et al., 2013), and other research has examined the influence of external distractors (Fried, 2008; Sana et al., 2013; Tesch et al., 2011), much less work has examined these two types of lapses together. Thus, one main goal was to examine rates of both mind-wandering and external distraction both in classes (during lectures) and while studying. Based on prior research it is likely that mind-wandering is more of an influence during class, but external distraction is more of an influence during studying (e.g., Unsworth, McMillan, et al., 2012). Another main goal of the current project was to examine different types of specific attentional disengagements. That is, we categorized disengagements into different subtypes of mind-wandering and external distraction in order to get a better sense of what types of mind-wandering are most likely and what different distractors are most prevalent. Given recent research suggesting that technology use is a major distractor in class, we expected that this would be one of the most common distractors reported. Another major goal of the current study was to examine individual differences in real-world disengagements of attention. As mentioned previously, both cognitive (cognitive abilities) and noncognitive (motivation and interest) person factors likely influence disengagements of attention in educational contexts. Thus, we examined whether these different factors predicted mind-wandering and external distraction overall, as well as predicting different subtypes of disengagements. Prior research has suggested that cognitive abilities (in particular WMC and attention control) are only related to certain types of disengagements, rather than to all types of disengagements (e.g., Kane et al., 2007; Kane et al., in press; Unsworth, McMillan, et al., 2012). Thus, it was expected that cognitive and noncognitive individual differences factors would be related to some forms of disengagement, but not necessarily to all forms of disengagement. A final major goal of the current study was to examine how these various factors (disengagements, cognitive abilities, motivation, and interest) would predict subsequent academic performance (end of term grades). To address these questions we a tested a large number of participants (*N* = 224) on several laboratory tasks to measure WMC, fluid intelligence, attention control, mind-wandering, and external distraction. During the laboratory session participants also completed questionnaires pertaining to their motivation to do well in class and their interest in the course materials for each of their classes. A subset of participants (*N* = 114) also agreed to carry diaries for a week in which they recorded attentional disengagements (mind-wandering and external distraction) they experienced each day in class or while studying. Finally, a subset of participants (*N* = 74) agreed to allow us to access their grades at the end of the term. By examining different types of attentional disengagements, cognitive abilities, and motivation and interest with a large sample of tasks and participants, we aimed to better elucidate the nature of attentional engagement and disengagement in educational contexts.

## Method

### Participants

A total of 224 participants (69% female) were recruited from the subject-pool at the University of Oregon. Participants were between the ages of 18 and 35 years (*M* = 19.87, *SD* = 3.05) and received course credit for their participation. Each participant was tested individually in a laboratory session lasting approximately 2 h. Two participants were excluded for failing to complete the laboratory session. Of the remaining 222 participants, 114 agreed to carry diaries for a week in which they recorded their attentional disengagements in class and while studying. We tested participants over two full academic quarters, using the end of the second quarter as our stopping rule for data collection with the aim of obtaining at least 100 individuals with complete diary data.

### Materials and procedure

After signing informed consent, all participants completed: operation span, symmetry span, reading span, Raven Advanced Progressive Matrices, letter sets, Sustained Attention to Response Task, antisaccade, and psychomotor vigilance task. All tasks were administered in the order listed above. Following the task, participants filled out a battery of questionnaires including a questionnaire asking them to list their classes for the term as well as their motivation and interest for each class. The other questionnaires were for a different project and not examined here. Finally, participants who were willing to participate in the diary portion of the study were given detailed instructions on the diaries.

### Laboratory tasks

#### Working memory capacity (WMC) tasks

##### Operation span (Ospan)

Participants solved a series of math operations while trying to remember a set of unrelated letters (F, H, J, K, L, N, P, Q, R, S, T, Y). Participants were required to solve a math operation and after solving the operation they were presented with a letter for 1 s. Immediately after the letter was presented the next operation was presented. Three trials of each list-length (3–7) were presented for a total possible of 75. The order of list-length varied randomly. At recall, letters from the current set were recalled in the correct order by clicking on the appropriate letters (see Unsworth, Heitz, Schrock, & Engle, 2005 for more details). Participants received three sets (of list-length 2) of practice. For all of the span measures, items were scored if the item was correct and in the correct position. The score was the proportion of correct items in the correct position.

##### Symmetry span (Symspan)

In this task participants were required to recall sequences of red squares within a matrix while performing a symmetry-judgment task. In the symmetry-judgment task participants were shown an 8 × 8 matrix with some squares filled in black. Participants decided whether the design was symmetrical about its vertical axis. The pattern was symmetrical half of the time. Immediately after determining whether the pattern was symmetrical, participants were presented with a 4 × 4 matrix with one of the cells filled in red for 650 ms. At recall, participants recalled the sequence of red-square locations in the preceding displays, in the order they appeared by clicking on the cells of an empty matrix (see Unsworth, Redick, Heitz, Broadway, & Engle, 2009 for more details). There were three trials of each list-length with list-length ranging from 2–5 for a total possible of 42. The same scoring procedure as Ospan was used.

##### Reading span (Rspan)

Participants were required to read sentences while trying to remember the same set of unrelated letters as Ospan. For this task, participants read a sentence and determined whether the sentence made sense or not (e.g., “The prosecutor’s dish was lost because it was not based on fact. ?”). Half of the sentences made sense while the other half did not. Nonsense sentences were made by simply changing one word (e.g., “dish” from “case”) from an otherwise normal sentence. Participants were required to read the sentence and to indicate whether it made sense or not. After participants gave their response they were presented with a letter for 1 s. At recall, letters from the current set were recalled in the correct order by clicking on the appropriate letters (see Unsworth et al., 2009 for more details). There were three trials of each list-length with list-length ranging from 3–7 for a total possible of 75. The same scoring procedure as Ospan was used.

#### Fluid intelligence (gF) tasks

##### Raven Advanced Progressive Matrices

The Raven is a measure of abstract reasoning (Raven, Raven, & Court, 1998). The test consists of 36 items presented in ascending order of difficulty (i.e., easiest-hardest). Each item consists of a display of 3 × 3 matrices of geometric patterns with the bottom right pattern missing. The task for the participant is to select among eight alternatives, the one that correctly completes the overall series of patterns. Participants had 10 min to complete the 18 odd-numbered items. A participant’s score was the total number of correct solutions. Participants received two practice problems.

##### Letter sets

In this task participants saw five sets of four letters, and participants were required to induce a rule that applies to the composition and ordering of four of the five letter sets (Ekstrom, French, Harman, & Dermen, 1976). Participants are then required to indicate the set that violates the rule. Following two examples, participants had 5 min to complete 20 test items. A participant’s score was the total number of items solved correctly.

#### Attention control (AC) tasks

##### Sustained Attention to Response Task (SART)

Participants completed a version of a Sustained Attention to Response Task (SART) with semantic stimuli adapted from McVay and Kane (2009, 2012). The SART is a go/no-go task where subjects must respond quickly with a key press to all presented stimuli except infrequent (11%) target trials. In this version of SART, word stimuli were presented in Courier New font size 18 for 300 ms followed by a 900-ms mask. Most of the stimuli (nontargets) were members of one category (animals) and infrequent targets were members of a different category (foods). The SART had 470 trials, 50 of which were targets. The dependent variables were accuracy for targets and each individual’s standard deviation of response time for go trials. Thought probes followed 60% of target trials.

##### Antisaccade

In this task (Kane, Bleckley, Conway, & Engle, 2001) participants were instructed to stare at a fixation point which was onscreen for a variable amount of time (200–2200 ms). A flashing white “=” was then flashed either to the left or right of fixation (11.33° of visual angle) for 100 ms. This was followed by a 50-ms blank screen and a second appearance of the cue for 100 ms making it appear as though the cue (=) flashed onscreen. Following another 50 ms blank screen the target stimulus (a B, P, or R) appeared onscreen for 100 ms followed by masking stimuli (an H for 50 ms and an 8 which remained onscreen until a response was given). All stimuli were presented in Courier New with a 12-point font. The participants’ task was to identify the target letter by pressing a key for B, P, or R (keys 1, 2, or 3 on the number keypad) as quickly and accurately as possible. In the prosaccade condition the flashing cue (=) and the target appeared in the same location. In the antisaccade condition the target appeared in the opposite location as the flashing cue. Participants received, in order, 10 practice trials to learn the response mapping, 10 trials of the prosaccade condition, and 50 trials of the antisaccade condition. The dependent variable was accuracy on the antisaccade trials. Thought probes followed 16% of antisaccade trials.

##### Psychomotor vigilance task (PVT)

In the psychomotor vigilance task (Dinges & Powell, 1985) participants were presented with a row of zeros on screen and after a variable amount of time the zeros began to count up in 1-ms intervals from 0 ms. The participants’ task was to press the spacebar as quickly as possible once the numbers started counting up. After pressing the spacebar the RT was left on screen for 1 s to provide feedback to the participants. Interstimulus intervals were randomly distributed and ranged from 1 to 10 s. The entire task lasted for 10 min for each individual (roughly 75 total trials). The dependent variable was the average reaction time for the slowest 20% of trials (Dinges & Powell, 1985). Thought probes followed 20% of trials.

### Thought probes

During the attention control tasks, participants were periodically presented with thought probes asking them to classify their immediately preceding thoughts. The thought probes asked participants to press one of five keys to indicate what they were thinking just prior to the appearance of the probe. Specifically, participants saw:

Please characterize your current conscious experience:

1. I am totally focused on the current task
2. I am thinking about my performance on the task or how long it is taking
3. I am distracted by sights/sounds or by physical sensations (hungry/thirsty)
4. I am zoning out/my mind is wandering
5. Other

These thought probes were based on those used by Stawarczyk, Majerus, Maj, Van der Linden, and D’Argembeau (2011) and Unsworth and McMillan (2014). During the instructions participants were given specific instructions regarding the different categories. Similar to prior research, response 3 was classified as external distraction and response 4 was classified as mind-wandering. Response 1 was considered as on-task thoughts, while response 2 was considered as task-related interference. Task-related interference refers to evaluative thoughts about the task or about task performance (i.e., “I’m not very good at this,” “This task is boring”, e.g., Sarason, Sarason, Keefe, Hates, & Shearin, 1986; Smallwood et al., 2004).

### Motivation and Interest Questionnaire

For each class participants were currently enrolled in they were asked to indicate on a scale from 1 to 6 how motivated they were to perform well in the class. They were also asked for each class to indicate on a scale from 1 to 6 how interested they were in the content of the course. Participants also indicated what grade they expected to receive in the course at the end of the term.

### Grades

In addition to the above measures we also obtained grades for each class at the end of the term via self-report from 74 participants.

### Diary

Participants were given a booklet and asked to keep a diary of their attention disengagements in class or while studying over the course of 1 week. Participants were told to indicate their various disengagements by writing a brief description of the disengagement and recording whether it occurred in class or while studying and to record what class they were in or what class they were studying for. Participants were encouraged to document the disengagements as soon as they happened or soon after they happened. Additionally, participants were instructed to classify each as either distraction or mind-wandering based on the following scheme.

1. Distraction – When task-irrelevant information captures your attention, thus keeping you from focusing on your task (i.e., your roommate’s cell phone keeps ringing)
2. Mind-wandering – When you find yourself lost in thoughts which are totally unrelated to a task (i.e., daydreaming in class)
3. Other

Each disengagement was classified as being due to distraction, mind-wandering, or other. Each disengagement was further classified based on the subclassification for each main type of disengagement. The subclassifications were based on our prior diary research (Unsworth, McMillan, et al., 2012), prior research examining mind-wandering and distraction in educational settings (e.g., importance of self-distraction with technology), and pilot research. All responses were checked by two raters to make sure that the descriptions of each error matched the classification provided by the participant. Inter-rater agreement was high (>95%), and disagreements were resolved. Participants were given detailed instructions about how to record responses in the diary and examples were provided to assist them.

## Results

The results are divided into three primary sections. In the first section, results from the diaries were examined in detail. In the second section, relations among the diary responses, cognitive ability measures, and the motivation and interest questionnaire were examined. In the final section, relations with academic performance were examined.

### Attentional disengagements

**Fig. 1:**
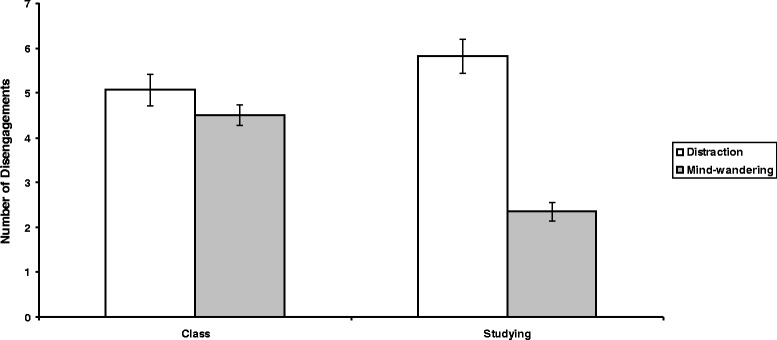
Number of attentional disengagements as a function of type of disengagement and context. *Error bars* represent one standard error of the mean

For each individual’s diary we counted the total number of reported disengagements as well as the total number of instances of distractions and total number of instances of mind-wandering for both in class and while studying. Overall, there was a total of 2433 attention disengagements reported across all participants. Of these, 1223 were instances of distraction and 1066 were instances of mind-wandering. Participants reported on average 21.34 (*SD* = 9.39) attention disengagements per week. Of these, 10.73 (*SD* = 6.12) were instances of distraction and 9.35 (*SD* = 4.24) were instances of mind-wandering. Next, we examined overall distractions and mind-wandering in class and while studying. Responses were submitted to a 2 (Disengagement: distraction versus mind-wandering) × 2 (Context: in class versus studying) repeated measures analysis of variance (ANOVA). There was a main effect of Disengagement, *F*(1, 113) = 43.45, *MSE* = 10.59, *p* < .001, partial *η* ^2^ = .28, suggesting that reports of distractions were more frequent than reports of mind-wandering. There was a main effect of Context, *F*(1, 113) = 7.84, *MSE* = 7.16, *p* = .006, partial *η* ^2^ = .07, with more disengagements in class (*M* = 9.58, *SD* = 4.92) than while studying (*M* = 8.18, *SD* = 5.27). Additionally, there was a Disengagement × Context interaction, *F*(1, 113) = 45.30, *MSE* = 5.27, *p* < .001, partial *η* ^2^ = .29. As seen in Fig. 1, there was no difference between distraction and mind-wandering reports in class, *t*(113) = 1.45, *p* = .15, *d* = .14, but there were more reports of distraction than mind-wandering while studying, *t*(113) = 9.67, *p* < .001, *d* = .99.

Next, we examined the different types of attentional disengagements in more detail. Of the total number of disengagements, 1983 attentional disengagements fell into one of 15 specific types of disengagements. The other disengagements were either relatively idiosyncratic disengagements or were not specified enough to be placed into one of the specific types. These included being distracted by the temperature of the classroom (typically too hot), distracted by the lighting in the room (lights flickering), as well as being distracted by other peoples’ odor (body odor, strong perfume or cologne). The major types of attention disengagements are listed in Table 1 in descending order of frequency. The most frequently occurring attention disengagements was being distracted by surrounding conversations and behaviors (such as someone leaving class early). These disengagements were followed by disengagements due to self-distraction (typically with technology such as texting or checking email, but also including doodling and tapping pencil/pen), mind-wandering and zoning out with no focus, mind-wandering about future social engagements (e.g., going to a party, going on a date), and mind-wandering because the course material/lecture was boring and not interesting. Other attentional disengagements included distracted by being tired, having to re-read something because of mind-wandering, mind-wandering about needing to do something in the future (e.g., planning), mind-wandering about future class performance (e.g., what grade is needed on the final to obtain a certain grade in the course), distracted because hungry, mind-wandering about a future meal, mind-wandering about prior class performance (e.g., worrying about a prior grade on an exam or assignment), distracted by class features (e.g., distracted by the PowerPoint, distracted by how the professor talks, moves their hands, or accidentally spits), distracted by current health (e.g., sick), and mind-wandering about the professor (e.g., thinking about why the professor is so weird or why they are so sweaty). Note, it is unlikely that the different types of failures are independent. That is, being distracted because you have hunger sensations is likely related to mind-wandering about a future meal. Likewise, being bored with the lecture content might result in daydreaming about future social engagements.

**Table 1:** Descriptive statistics and rankings of most frequent everyday attention disengagements

Attentional disengagement	*M*	*SD*	Sum	Range
1. Distracted surrounding conversation/behavior	5.11	3.77	582	24
2. Self-distraction	2.54	2.41	290	9
3. Zoning out	2.54	2.21	289	11
4. Mind-wandering future social engagement	1.19	1.49	136	8
5. Mind-wandering bored disinterested	1.17	1.43	133	7
6. Distracted tired	1.06	1.33	121	6
7. Mind-wandering had to re-read	0.97	1.22	111	5
8. Mind-wandering about what to do	0.64	0.93	73	4
9. Mind-wandering about future class performance	0.60	0.98	68	5
10. Distracted hungry	0.39	0.72	45	4
11. Mind-wandering about future meal	0.25	0.64	29	4
12. Mind-wandering about prior class performance	0.25	0.90	29	7
13. Distracted by class features	0.25	0.56	29	3
14. Distracted by current health	0.24	0.61	27	4
15. Mind-wandering about professor	0.19	0.46	22	2

### Relations among diary responses, cognitive abilities, and motivation and interest

Next, we examined the relations among the laboratory cognitive ability measures of WMC, fluid intelligence, attention control, as well as self-reports of mind-wandering and external distraction during the attention control tasks. Descriptive statistics for the measures are shown in Table 2. As can be seen in Table 2, the measures had generally acceptable values of internal consistency and most of the measures were approximately normally distributed with values of skewness and kurtosis under the generally accepted values (i.e., skewness < 2 and kurtosis < 4). Correlations among the cognitive ability measures, shown in Table 3, were weak to moderate in magnitude with measures of the same construct generally correlating stronger with one another than with measures of other constructs, indicating both convergent and discriminant validity within the data.

**Table 2:** Descriptive statistics and reliability estimates for the cognitive ability measures

Measure	M	SDS	Skew	Kurtosis	Reliability
Ospan	58.19	11.33	−1.01	.73	.76
Symspan	30.08	6.28	−.48	−.03	.65
Rspan	56.40	11.62	−.88	.73	.77
Raven	8.55	2.93	−.20	−.03	.67
LS	10.34	2.95	−.10	−.30	.68
Anti	.49	.13	.69	.07	.95
SARTacc	.56	.15	.13	−.38	.85
SARTSD	146.17	39.70	.56	1.29	.83
PVT	540.62	117.98	1.28	1.80	.78
AntiMW	.18	.24	1.68	2.59	—
SARTMW	.22	.19	1.09	1.04	—
PVTMW	.18	.18	1.16	1.06	—
AntiED	.11	.16	2.15	5.47	—
SARTED	.14	.12	.92	.48	—
PVTED	.13	.15	2.04	5.95	—

Ospan = operation span; Rspan = reading span; Symspan = symmetry span; Raven = Raven Advanced Progressive Matrices; LS = letter sets; NS = Anti = antisaccade; SARTacc = accuracy on Sustained Attention to Response Task; SARTSD = standard deviation of response time on the Sustained Attention to Response task; PVT = psychomotor vigilance task; ED = external distraction; MW = mind-wandering

**Table 3:** Correlations among the cognitive ability measures

Measure	1	2	3	4	5	6	7	8	9	10	11	12	13	14	15
1. Ospan	—														
2. Symspan	0.33	—													
3. Rspan	0.65	0.35	—												
4. Raven	0.33	0.36	0.28	—											
5. LS	0.25	0.33	0.21	0.34	—										
6. Anti	0.25	0.17	0.23	0.26	0.26	—									
7. SARTacc	0.08	0.12	0.09	0.24	0.14	0.19	—								
8. SARTSD	−0.05	−0.07	−0.11	−0.16	−0.17	−0.25	−0.11	—							
9. PVT	−0.08	−0.10	−0.09	−0.20	−0.14	−0.27	−0.23	0.19	—						
10. AntiMW	0.01	−0.08	−0.04	−0.14	−0.02	−0.16	−0.18	0.10	0.04	—					
11. SARTMW	−0.11	−0.14	−0.15	−0.25	−0.19	−0.25	−0.23	0.30	0.18	0.47	—				
12. PVTMW	−0.02	−0.07	−0.08	−0.08	0.08	−0.06	−0.21	0.06	0.11	0.33	0.43	—			
13. AntiED	−0.06	−0.10	−0.06	0.01	−0.04	−0.01	−0.04	−0.07	0.10	−0.09	0.00	0.09	—		
14. SARTED	−0.04	−0.04	−0.06	0.00	0.05	0.07	−0.09	−0.07	0.04	−0.03	−0.10	−0.04	0.38	—	
19. PVTED	−0.05	−0.08	0.01	0.06	−0.05	−0.02	−0.07	−0.11	0.04	−0.12	−0.12	−0.21	0.28	0.38	—

Ospan = operation span; Rspan = reading span; Symspan = symmetry span; Raven = Raven Advanced Progressive Matrices; LS = letter sets; NS = Anti = antisaccade; SARTacc = accuracy on Sustained Attention to Response Task; SARTSD = standard deviation of response time on the Sustained Attention to Response task; PVT = psychomotor vigilance task; ED = external distraction; MW = mind-wandering

**Fig. 2:**
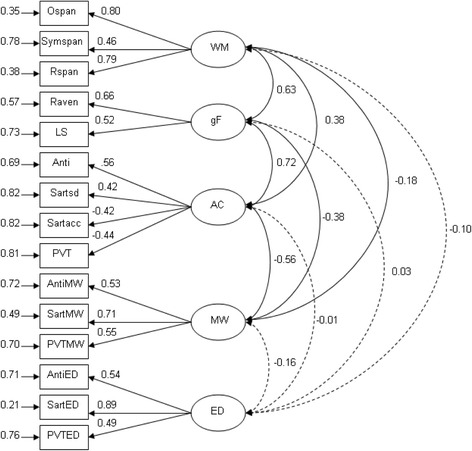
Confirmatory factor analysis model for working memory capacity (WMC), fluid intelligence (gF), attention control (AC), mind-wandering during the laboratory tasks (MW), and external distraction during the laboratory tasks (ED). Paths connecting latent variables (*circles*) to each other represent the correlations between the constructs and the numbers from the latent variables to the manifest variables (*squares*) represent the loadings of each task onto the latent variable. Solid paths are significant at the *p* < .05 level, whereas dashed paths are not significant. Ospan = operation span; Rspan = reading span; Symspan = symmetry span; Raven = Raven Advanced Progressive Matrices; LS = letter sets; Anti = antisaccade; Sartsd = standard deviation of response time on the Sustained Attention to Response task; Sartacc = accuracy on Sustained Attention to Response Task; PVT = psychomotor vigilance task; AntiMW = mind-wandering during antisaccade; SartMW = mind-wandering during SART; PVTMW = mind-wandering during psychomotor vigilance task; AntiED = external distraction during antisaccade; SartED = external distraction during SART; PVTED = external distraction during psychomotor vigilance task

We next used confirmatory factor analysis to examine a measurement model of all of the cognitive ability measures to determine how each of the putative factors were related to one another. Separate factors were formed for WMC, fluid intelligence, attention control, mind-wandering, and external distraction. All of the factors were allowed to correlate. This model tests the extent to which different measures can be grouped into separate yet correlated factors, and examines the latent correlations among the factors. The fit of the model was acceptable, *χ* ^2^ (80) = 103.03, *p* = .043, RMSEA = .04, SRMR = .06, NNFI = .96, CFI = .97, suggesting that the specified model provided a good description to the underlying pattern of data. Shown in Fig. 2 is the resulting model. As can be seen each of the tasks loaded significantly on their respective constructs and most of the latent constructs were moderately correlated with one another. Specifically, consistent with prior research (Kane et al., 2016; McVay & Kane, 2012; Unsworth & McMillan, 2014), WMC, fluid intelligence, attention control, and mind-wandering were all correlated with one another. The only factor that did not correlate with the other factors was external distraction. The lack of a relation of this factor with the other factors is inconsistent with prior research (i.e., Unsworth & McMillan, 2014). Possible reasons for the discrepant findings will be discussed in the “Discussion.” Overall, most of the laboratory cognitive ability measures were related to one another and latent factors could be constructed from the underlying data.

Next we included overall distraction and mind-wandering responses from the diaries into the model (as manifest variables) to see if they would relate to the cognitive ability measures and to motivation and interest in classes. Note that given that many of the distributions of attention disengagements were positively skewed we applied a square root transformation to each disengagements and correlations were based on the transformed values. The fit of the model was acceptable, *χ* ^2^(120) = 78.35, *p* = .99, RMSEA = .00, SRMR = .05, NNFI = 1.14, CFI = 1.0. Note, motivation and interest in classes were not significantly related to any of the cognitive ability measures, but were correlated with one another (*r* = .45). Furthermore, consistent with prior research mind-wandering and distraction were correlated (*r* = .36; Unsworth, Brewer, et al., 2012). Shown in Table 4 are the resulting correlations.^1^ Surprisingly, the only significant correlation with the total number of instances of distraction was a positive correlation with fluid intelligence. For mind-wandering the only significant correlation was a negative correlation with MW during the attention control tasks. Both of these relations are in directions opposite of what would be expected.

**Table 4:** Latent correlations of attentional disengagements with working memory capacity (WMC), fluid intelligence (gF), attention control (AC), laboratory mind-wandering (MW), laboratory external distraction (ED), motivation in classes (Mot), and interest in classes (Inter)

Attentional disengagements	WMC	gF	AC	MW	ED	Mot	Inter
Distraction total	.16	.36^a^	.20	−.18	.12	.02	.04
Mind-wandering total	.12	.17	.19	−.34^a^	.03	.01	−.06
1. Distracted surrounding conversation/behavior	.06	.18	.25^a^	−.36^a^	.03	.08	.09
2. Self-distraction	.08	.42^a^	.04	.02	.01	−.01	−.24^a^
3. Zoning out	.14	.13	.09	.08	−.10	.03	−.04
4. Mind-wandering future social engagement	.06	−.06	.05	−.27^a^	.09	.05	.04
5. Mind-wandering bored disinterested	−.20^a^	−.33^a^	−.22	.22^a^	−.03	−.21^a^	−.14
6. Distracted tired	−.03	−.20	−.17	.15	.31^a^	−.05	−.01
7. Mind-wandering had to re-read	−.01	.03	−.09	−.14	.00	−.05	.03
8. Mind-wandering about what to do	.04	−.10	.07	−.29^a^	−.07	.07	.04
9. Mind-wandering about future class performance	.03	−.21	.06	.00	.04	.22^a^	.18^a^
10. Distracted hungry	.06	−.09	.04	−.14	.01	−.05	.02
11. Mind-wandering about future meal	.01	.05	.01	−.13	−.11	.00	.02
12. Mind-wandering about prior class performance	−.28^a^	−.26^a^	.00	−.17	.20	.02	.11
13. Distracted by class features	−.14	−.23	−.08	.23^a^	−.04	−.17	−.15
14. Distracted by current health	−.15	−.29	−.19^a^	−.07	.15	.17	.13
15. Mind-wandering about professor	−.09	.11	.08	−.14	−.16	−.05	−.11

^a^ *p* < .05

To better examine these overall relations and to get a better sense of the data we next included the 15 specific disengagements (total number of each for each participant) into the model (as manifest variables) to see how cognitive abilities and motivation and interest in classes would relate with each type of disengagement. The fit of the model was acceptable, *χ* ^2^(250) = 191.51, *p* = .99, RMSEA = .00, SRMR = .05, NNFI = 1.17, CFI = 1.0. Shown in Table 4 are the resulting correlations. As can be seen, WMC was negatively related to mind-wandering while bored or disinterested in the current class and WMC was negatively correlated with mind-wandering about prior class performance. Fluid intelligence was positively related to self-distraction, but negatively related to mind-wandering while bored or disinterested in the current class, mind-wandering about prior class performance, and distracted by current health. Thus, the positive relation between fluid intelligence and distraction was largely driven by the fact that high-fluid-intelligence students seem to be more likely to distract themselves (mostly via technology) during lecture and while studying. The only significant correlation involving attention control was a positive correlation with distraction due to surrounding conversations and behavior. Self-reports of mind-wandering during the attention control tasks were negatively related to mind-wandering about future social engagements and mind-wandering about what to do (i.e., planning), but were positively related to mind-wandering while bored or disinterested in the current class and distracted by class features. Thus, it seems to be the case that self-reports of mind-wandering during challenging laboratory tasks may be indexing a particular type of mind-wandering. Self-reports of external distraction during the attention control tasks were only positively correlated with distraction due to being tired. Motivation to do well in class was negatively related to mind-wandering while bored or disinterested in the current class and positively related to mind-wandering about future class performance. Finally, interest in the content of the class was negatively related to self-distraction, but positively related to mind-wandering about future class performance. Thus, those individuals who were motivated to do well and interested in the content of the class were less likely to self-distract themselves, less likely to mind-wander because they were bored, and were more likely to think about future class performance.

Note, given the large number of correlations that were examined, it is likely that some of these relations are a result of a Type-I error. Although most latent variable studies do not correct for multiple comparisons (see Cribbie, 2000, 2007; Smith & Cribbie, 2013 for discussions), we examined whether the significant correlations would survive corrections. Using a strict Bonferroni correction suggested only two correlations (laboratory mind-wandering to distraction via surrounding conversations/behavior and fluid intelligence to self-distraction) would remain significant. Taking into account correlations among the parameters would have likely resulted in additional correlations being deemed significant (Smith & Cribbie, 2013). Using Benjamini and Hochberg’s (1995) false discovery rate (suggested by Cribbie, 2007 for use with structural equation modeling) suggested that six correlations (laboratory mind-wandering to distraction via surrounding conversations/behavior, fluid intelligence to self-distraction, working memory to mind-wandering about prior class performance, laboratory mind-wandering to mind-wandering about what to do, laboratory external distraction to tired, and interest to self-distraction) would remain significant. Additionally, Kane et al. (in press) recently examined relations between laboratory cognitive ability measures and laboratory mind-wandering (similar to the current assessments) with everyday mind-wandering in a large sample of participants. Similar to the current study, Kane et al. examined a large number of relations. In order to deal with potential Type-I errors they set their alpha level to *p* = .005. Doing the same for the current results leads to similar correlations being deemed significant as Benjamini and Hochberg’s false discovery rate correction. Thus, it is likely that not all of the relations that were found are robust. Importantly, several of the relations between the attentional disengagements and the laboratory measures cut across several different measures (for example, mind-wandering while bored or disinterested correlated with a number of variables) suggesting a likely reliable relation. Future research is needed to further examine relations between everyday attentional disengagements in educational contexts and cognitive abilities and person factors measured in the laboratory.

### Academic performance

The final set of analyses focused on academic performance. Recall that a subset of participants (*N* = 74) agreed to provide their grades for each class at the end of the term. Average grade point average (GPA) was 3.14 (*SD* = .69). The first set of analyses examined whether academic performance in a given class was associated with the number of attention disengagements experienced in that class, motivation to do well in that class, and interest in the content of that class. Note, because not all participants reported taking the same number of classes, we used linear mixed models to analyze the data. Linear mixed models are an extension of the general linear model in which both fixed and random effects are included. Thus, they are similar to mixed ANOVA but offer advantages over traditional mixed ANOVAs in terms of more power and the ability to handle unbalanced designs and missing data (e.g., Kliegl, Wei, Dambacher, Yan, & Zhou, 2011). In the models, attentional disengagements (overall and broken down by distraction and mind-wandering), motivation, and interest were entered as fixed factors, and subjects were entered as random factors. Examining attentional disengagements suggested no relation between number of disengagements and subsequent academic performance, *t* = –1.47, *p* = .144 (*b* = –.018, *SE* = .012). Similar results were found when examining only distractions, *t* = −1.19, *p* = .24 (*b* = −.020, *SE* = .016), and examining only mind-wandering, *t* = −.82, *p* = .41 (*b* = −.021, *SE* = .026). Examining motivation to do well in the class suggested a positive relation between motivation and subsequent academic performance, *t* = 2.80, *p* = .005 (*b* = .154, *SE* = .054). Examining interest in the content of the class suggested a slight positive relation between interest and subsequent academic performance, *t* = 1.89, *p* = .06 (*b* = .066, *SE* = .034). Given a strong correlation between motivation and interest in these within subject analyses, *r*(880) = .50, the next model examined motivation and interest together, along with their interaction. This model suggested a positive relation between motivation and grades, *t* = 2.32, *p* = .021 (*b* = .288, *SE* = .124), but no relation with interest, *t* = 1.60, *p* = .112 (*b* = .244, *SE* = .153), and no interaction, *t* = −1.45, *p* = .15 (*b* = −.042, *SE* = .029). Thus, motivation to do well in a particular class was a positive predictor of subsequent academic performance in that class.

Next, we examined whether cognitive abilities, motivation and interest in classes, and attentional disengagements would predict academic performance at the individual differences level. Note for these analyses given the reduced sample size we created factor composites for each cognitive ability measure rather than relying on confirmatory factor analysis. As shown in Table 5, individual differences in fluid intelligence, mind-wandering during the attention control tasks, and motivation to do well in class all predicted average GPA for that term. Note in this subset of participants, mind-wandering during the attention control tasks and motivation were correlated (*r* = −.34, *p* = .004), mind-wandering during the attention control tasks and fluid intelligence were correlated (*r* = −.32, *p* = .006), but fluid intelligence and motivation were not correlated *r* = −.02, *p* = .86). To examine these relations in more detail we submitted all three variables to a simultaneous regression predicting the estimates of GPA. Collectively, these three variables accounted for 20% of the variance in GPA. As seen in Table 6, both fluid intelligence and motivation to do well accounted for unique variance in GPA, but laboratory mind-wandering reports did not. These results are consistent with prior research suggesting that individual differences in intelligence and motivation are important predictors of overall GPA (Kuncel, Hezlett, & Ones, 2004; Spinath, Spinath, Harlaar, & Plomin, 2006; Steinmayr & Spinath, 2009).

**Table 5:** Correlations of average grade point average (GPA) with working memory capacity (WMC), fluid intelligence (gF), attention control (AC), mind-wandering self-reports (MW), external distraction self-reports (ED), motivation in classes, interest in classes, total number of distraction failures from diaries, and total number of mind-wandering failures from diaries

Measure	GPA
WMC	.02
gF	.31^a^
AC	.21
MW	−.26^a^
ED	−.17
Motivation	.31^a^
Interest	.14
Distraction total	.14
Mind-wandering total	.14

^a^ *p* < .05

**Table 6:** Simultaneous regression predicting grade point average (GPA)

Variable	*B*	*T*	*sr* ^*2*^	*R* ^*2*^	*F*
gF	.29	2.57^a^	.076		
MW	−.06	−.53	.00		
Motivation	.29	2.56^a^	.075	.20	5.75^b^

^a^ *p* < .05, ^b^ *p* < .01. MW mind-wandering self-reports

## Discussion

In a unique approach to studying attention disengagements in educational contexts, we combined diary methods with laboratory assessments of various cognitive abilities and questionnaires indexing motivation and interest in current classes in a large sample of participants. In a sample of undergraduate students we found that students experience a number of different attentional disengagements both during lecture and while studying throughout the course of a typical week. Specifically, we found that distractions were slightly more frequent than mind-wandering, and more disengagements occurred in class than while studying. Note, a potential issue with this analysis is that it assumes that equal amounts of time are spent in class and while studying. Unfortunately, we did not have a measure of how much time was spent in each, so the main effect of context may simply reflect the fact that more time is spent studying than in class. Future research is needed to better examine how amount of time in each influences the results. Additionally, we found that these factors interacted such that rates of distraction were similar in both class and while studying, but mind-wandering was more frequent during lecture than while studying. This reduction in mind-wandering during study could reflect the fact that during study students frequently interrupt themselves and attempt to multitask via self-distractions with technology (Calderwood et al., 2014). Thus, during study participants may be directing their attention to other external activities (checking email, checking Facebook, listening to music, etc.) rather than engaging in mind-wandering. These results are consistent with prior diary work suggesting that distraction while studying and mind-wandering during lectures are two potent forms of attentional disengagements (Unsworth, McMillan, et al., 2012). Furthermore, although much prior research has examined mind-wandering or distraction in educational contexts, the current work demonstrates the importance of examining these two types of lapses together to get a better sense of their relative frequencies and how they change in different contexts (in class or while studying). Collectively, the current results replicate and extend prior research by demonstrating that mind-wandering and distraction are frequently occurring disengagements of attention for students in different educational contexts (Brown, 1927, Cameron & Giuntoli, 1972; Fried, 2008; Geerligs, 1995; Lindquist & McLean, 2011; Schoen, 1970; Tesch et al., 2011; Sana et al., 2013; Shelton et al., 2009; Unsworth, McMillan, et al., 2012; Wammes, Boucher, et al., 2016).

Examining various subtypes of attentional disengagements suggested a number of interesting findings. For example, by far the most common attentional disengagement was distraction due to surrounding conversations and behaviors. Out of the 1983 disengagements that could be classified, this type of distraction represented 29.3% of all attentional disengagements. Importantly this type of distraction occurred both in class and while studying. Thus, not only are students distracted while trying to study in a busy coffee shop, dorm, or student union, but distractions are also prevalent during the lectures themselves. Consistent with prior research another prominent distraction was self-distraction typically associated with technology (e.g., texting, checking email, checking Facebook, etc.; Fried, 2008; Hollis & Was, 2016; Junco, 2012; Ravizza et al., 2017; Risko et al., 2013; Sana et al., 2013; Wood et al., 2012). Self-distractions represented 14.6% of the reported attentional disengagements. Other important disengagements were zoning out (14.6%), mind-wandering about future social engagements (6.9%), and mind-wandering because the lecture content or study material was disinteresting or boring (6.7%). Participants also reported being distracted because they were tired (6.1%), having to re-read something because they found themselves mind-wandering (5.6%), mind-wandering about something they needed to do (3.7%), and mind-wandering about future class performance (3.4%; typically participants indicated they were worried about an upcoming exam). Less frequently occurring attentional disengagements included being distracted because they were hungry (2.3%), mind-wandering about a future meal (1.5%), mind-wandering about prior class performance (1.5%; typically associated with worry because of poor prior exam performance), distracted by features of the class (1.5%), distracted by current health (1.4%; typically because the participants mentioned being sick), and mind-wandering about the professor (1.1%). Collectively these results suggest that not only are students distracted and prone to mind-wandering in class and while studying, but there is a large range of things that cause distraction and a large range of topics students tend to mind-wander about. Furthermore, these results are broadly consistent with prior research suggesting that the content of mind-wandering is associated with current concerns (Klinger, 2013; Klinger & Cox, 1987–1988), with some thoughts being more deliberate and others being more spontaneous (Giambra, 1995; Seli, Risko, et al., 2016; Wammes, Seli, et al., 2016), and with much of mind-wandering being associated with a prospective bias related to goals of the individual, whether the goal is to do well in a class or get a cheeseburger after class because you are hungry (Andrews-Hanna et al., 2013; Baird, Smallwood, & Schooler, 2011; Stawarczyk et al., 2011).

Examining individual differences in attentional disengagements suggested that everyday mind-wandering and distraction were positively correlated (*r* = .36) and at a similar magnitude as prior research (*r* = .43; Unsworth, Brewer, et al., 2012). Those individuals who experience one type of attentional disengagements are also likely to experience other attentional disengagements. Examining relations with cognitive abilities assessed in the laboratory suggested a number of interesting findings. Specifically, the total number of distractions was positively related to fluid intelligence, whereas the total number of mind-wandering failures was negatively related to mind-wandering rates from the laboratory tasks. Both of these relations are opposite of what would be expected. However, an examination of the subtypes of disengagements suggests possible reasons for the results. For example, fluid intelligence was positively related to self-distractions, but tended to be more negatively correlated with other distractors (in particular distracted by current health). Thus, in the current data higher intelligence individuals were more likely to distract themselves in class and while studying than less intelligent individuals. This could be due to high-intelligence individuals being better able to multitask than low-intelligence individuals (Redick et al., 2016) as well as high-intelligence individuals relying on more effective study strategies than low-intelligence individuals (Hartwig & Dunlosky, 2012), which would allow them to potentially disengage more often during lectures and/or while studying. Mind-wandering rates from the laboratory tasks were negatively associated with mind-wandering rates from the diaries, and this was reflected in negative associations between the laboratory mind-wandering factor and mind-wandering about future social engagements and mind-wandering about what one needed to do. However, positive relations between laboratory mind-wandering and mind-wandering because one was bored and disinterested and being distracted by class features were also found. Thus, there were clear differences between mind-wandering in the laboratory and mind-wandering in daily life (Kane et al., in press). This suggests that mind-wandering from the laboratory tasks is likely indexing more spontaneous and/or unintentional mind-wandering, whereas some of the diary responses are associated with more deliberate/intentional mind-wandering (in particular with a prospective bias). This is consistent with recent work suggesting that mind-wandering in many laboratory tasks reflects more unintentional than intentional mind-wandering especially when the laboratory tasks are demanding (Seli, Risko, et al., 2016). This makes sense given that mind-wandering in the laboratory was assessed during three demanding attention control tasks. Consistent with this notion is the finding that cognitive abilities (WMC and fluid intelligence) assessed in the laboratory were negatively related to mind-wandering due to boredom and disinterest in the topic material as well as negatively related to mind-wandering about prior class performance (personal concern). This suggests that lapses of attention associated with more spontaneous mind-wandering due to personal concerns is related to lower cognitive abilities (McVay & Kane, 2010), but that more deliberate mind-wandering associated with prospection is not necessarily related to cognitive abilities (McVay, Unsworth, McMillan, & Kane, 2013). Clearly, more work is needed to better examine the notion that various types of mind-wandering are differentially related in and out of the laboratory (see also Kane et al., in press).

Similarly, external distraction assessed in the laboratory was only related to distractions due to being tired. As noted previously, this result is inconsistent with prior research which has shown that external distraction and mind-wandering in the laboratory are sometimes positively correlated (e.g., Unsworth & McMillan, 2014). One reason for these discrepant results could be due to how participants were tested in each study. In Unsworth and McMillan (2014), participants were tested in groups in one large room. Thus, there was ample opportunity for a number of distractions (i.e., the experimenter talking with other participants, other participants, moving around, participants arriving and leaving at different times, etc.). However, in the current study participants were tested alone in a quiet room (see also Stawarczyk et al., 2011, Robison & Unsworth, 2015 quiet condition). Thus, there were likely fewer external distractions present. In this case the primary distractors were likely interoceptive sensations such as feeling hot/cold, hungry/thirsty, tired, or other physical sensations (Stawarczyk et al., 2011). As such, this suggests that depending on the context, mind-wandering and external distraction assessed in the laboratory are likely indexing particular types of mind-wandering (unintentional mind-wandering during demanding tasks) and particular types of distraction (interoceptive sensations when tested in a quiet well-lit room) which then influences their relation with other cognitive abilities (Robison & Unsworth, 2015) and with everyday attentional disengagements.

Examining relations between attentional disengagements and motivation and interest suggested that participants who were motivated to perform well in their classes tend to mind-wander less when they were bored or disinterested with the course material and tended to mind-wander more about future class performance (such as thinking about an upcoming exam). Participants who reported being interested in their classes were less likely to self-distract themselves and similar to highly motivated individuals, were more likely to mind-wander about future class performance than individuals who were less interested in their classes. Thus, not only were cognitive abilities associated with individual differences in everyday mind-wandering and distraction, but other person variables, such as motivation to do well and interest in classes, were also predictive of certain types of attentional disengagements.

Examining end of the term grades suggested that classes that were associated with higher motivation ratings were also associated with higher grades. Additionally, replicating prior research, we found that intelligence (here, fluid intelligence) and motivation successfully predicted end of the term grades (Kuncel et al., 2004; Spinath et al., 2006; Steinmayr & Spinath, 2009). Mind-wandering assessed in the laboratory was also predictive of end of the term grades, but this relation was primarily due to shared variance with fluid intelligence and motivation. Once all three measures were examined together, only fluid intelligence and motivation accounted for unique variance in end of the term GPA. Interestingly, none of the everyday attentional disengagements from the diaries were correlated with GPA. The lack of relations may be due to the fact that our only measure of academic performance was end of the term GPA which might not be sensitive to in-the-moment disengagements. Indeed, Wammes, Seli, et al. (2016) found that mind-wandering during lectures was related to exam and quiz scores, but not to overall GPA. Given that students can study for exams outside of class, and many final grades are based on a combination of quizzes, exams, and final projects, it is possible that in-the-moment attentional disengagements lead to poor learning and retention, but students engage in other activities to obtain better grades. Indeed, an interesting finding from the current study was that high-fluid-intelligence individuals tended to self-distract more than low-fluid-intelligence individuals. Yet, high-fluid-intelligence individuals also tended to get better grades than low-fluid-intelligence individuals. Thus, these high-fluid-intelligence individuals may deliberately disengage and self-distract themselves knowing that they can obtain the desired grade by studying harder or putting extra effort into final projects.

### Relations to theories of off-task thoughts and activities

The current results are broadly consistent with a number of theories of off-task thoughts and activities. For example, Kanfer and Ackerman (1989) extended Kahneman’s (1973) model of attention to include not only motivational factors, but also included the notion that attention could be allocated to off-task thoughts. Specifically, Kanfer and Ackerman (1989; see also Kanfer, 1990) suggested depending on motivation and effort levels, attention may be allocated to the primary task at hand (taking notes) or may be allocated to off-task activities. If motivation is high (and individuals think that they can succeed), then attention is allocated to the primary task leading to task engagement. However, if motivation is low to perform the primary task, then attention can be allocated to off-task activities (daydreaming, self-distractions), leading to task disengagement. The current findings that motivation and interest are negatively related to self-distractions and mind-wandering because of boredom or disinterest in the material, but positively related to mind-wandering about future class performance is certainly in line with this reasoning suggesting important interactions between motivation and off-task thinking/task disengagement.

Likewise, Kanfer and Ackerman’s (1989) theory suggests that individual differences in cognitive abilities (working memory, intelligence, attention control) are indicative of individual differences in overall capacity with some individuals having more capacity than others. By this account, some individuals should have enough capacity to engage in both on-task and off-task activities (i.e., multitasking). The finding that fluid intelligence was positively related to self-distractions and to overall GPA is consistent with this notion. That is, high-fluid-intelligence individuals may have had more capacity to stay partially engaged while at the same time engaging in self-distraction activities and still perform well compared to low-fluid-intelligence individuals. This notion is consistent with the context-regulation theory of mind-wandering which suggests that mind-wandering is determined, in part, by the current context and the extent to which demands on attention are high or low (Smallwood, 2013; Smallwood & Andrews-Hanna, 2013). When attentional demands are high, mind-wandering needs to be suppressed via executive resources to ensure task engagement. In these situations, mind-wandering is thought to occur due to a failure of executive resources to keep attention engaged and focused on the primary task (Kane & McVay, 2012; McVay & Kane, 2009; Thomson, Besner, & Smilek, 2015). However, when attentional demands are low (or are perceived to be low), attention can be allocated to off-task thoughts that may be thought to be more important than the primary task (e.g., mind-wandering about something important you need to do after class). High-cognitive-ability individuals may be better suited to control their attention than low-cognitive-ability participants to ensure that when attentional demands are high, attention is properly allocated on the primary task and internal and internal distractors are prevented from capturing attention. This would result in negative relations between cognitive abilities and attentional disengagements in situations where demands for attention are high (see also Kane et al., 2007; Kane et al., in press). However, when attentional demands are low, high- and low-cognitive-ability participants may have enough capacity to allocate attention to off-task activities resulting in no relations between cognitive abilities and everyday attentional disengagements. Other times when attentional demands are low, high-cognitive-ability participants may be better able to allocate their attention to off-task thoughts or to divide their attention between on-task and off-task thoughts than low-cognitive-ability participants (e.g., Rummel & Boywitt, 2014). This would result in a positive relation between cognitive abilities and attentional disengagements as was seen for the relation between fluid intelligence and self-distractions. Thus, there are likely important interactions between individual differences in various cognitive abilities, task demands, and off-task thinking/task disengagement.

Finally, within this framework it is possible that arousal levels may influence the extent to which attention is disengaged from the primary task. For example, according to Kahneman’s (1973) model, when arousal levels are low, less attention is allocated to the primary task leading to less overall focus, and potentially a greater susceptibility to salient internal or external distractors (see also Lenartowicz et al., 2013). Thus, in these situations attention could be disengaged from the primary task and allocated to irrelevant sources of information involuntarily. Furthermore, individuals who are underaroused (due to a boring task/situation or due to low tonic arousal levels) or experience frequent fluctuations in arousal would be more likely to have involuntary attentional disengagements (Unsworth & Robison, in press) such as mind-wandering more when bored or disinterested in the material (see Eastwood, Frischen, Fenske, & Smilek, 2012 for an attentional account of boredom). That is, in situations where attention is supposed to be focused on the primary task (such as listening to a lecture and taking notes), but the individual is underaroused and bored with the material, then potent internal or external distractors are more likely to hijack attention away from the primary task.

### Limitations and future directions

Before concluding we should acknowledge a number of limitations of the current study. First, it should be acknowledged that diary responses reflect relatively coarse measures of attentional disengagements in everyday life. That is, despite the strengths of using diary methods to assess everyday attentional disengagements, there are also clear limitations with these types of studies. For example, given that diary methods require both prospective and retrospective memory and a heavy dependence on meta-awareness it is clear that not all disengagements will be reported and not all disengagements will be reported entirely accurately or in a timely fashion. Additionally, unlike probe caught methods, diary methods likely underestimate instances of mind-wandering when participants are unaware that they are mind-wandering. As noted by Reason and Lucas (1984) diaries “serve a valuable function as wide-gauge trawl nets, picking up the more salient types of lapse” (p. 56). Furthermore, unlike most probe-caught methods, diaries allow and encourage participants to completely describe each type of disengagements resulting in richer description. Thus, despite limitations, diary methods are particularly useful (especially when combined with laboratory measures and individual differences analyses) in obtaining more naturalistic data.

Second, as mentioned previously we did not specifically ask participants to note whether a particular disengagements was intentional or unintentional. Given the importance of examining potential differences between intentional versus unintentional disengagements (in particular, mind-wandering; Giambra, 1995; Seli, Risko, et al., 2016; Seli, Wammes, et al., 2016; Wammes, Seli, et al., 2016) it would have been beneficial to examine deliberate disengagements of attention versus more spontaneous and unintentional disengagements and how these are related to individual differences in cognitive abilities, motivation, interest, and academic performance. Future research should more finely measure and examine potential similarities and differences between intentional and unintentional disengagements not only for mind-wandering (e.g., Wammes, Seli, et al., 2016), but also for various distractions (especially self-distractions).

Finally, as noted above, our only measure of academic performance was end of the term grades. Given that participants participated for 1 week throughout the term it was not possible to tie attentional disengagements to quiz or exam scores. Future research should more thoroughly examine the relation between various attentional disengagements (both in class and while studying) and academic performance (quizzes, exams, final grades) by tracking participants throughout an entire term rather than at random points throughout the term. This would allow for a better assessment of attentional disengagements at important points during the term (before or after exams) and better examine whether the type and frequency of attentional disengagements are salient predictors of performance.

## Conclusions

The current study demonstrated that undergraduate students are susceptible to a number of different types of attentional disengagements that occur both in class and while studying. These disengagements range from being distracted by surrounding conversations, to mind-wandering because the current lecture is boring, to worrying about future exam performance. These everyday attentional disengagements were shown to be related to important cognitive abilities such as working memory capacity, attention control, and fluid intelligence. Furthermore, mind-wandering and external distraction assessed during demanding laboratory tasks were related to everyday attention disengagements in some situations. Motivation to do well and overall interest in class similarly predicted some types of attentional disengagements. Finally, academic performance (end of the term GPA) was related to motivation and fluid intelligence, but not to everyday attentional disengagements. The current results provide important evidence suggesting that undergraduate students are prone to mind-wandering and distraction in educational contexts with some students being more susceptible to certain types of attentional disengagements than others.

## Footnotes

1 On average students were enrolled in 4.07 (*SD* = .82) classes. The number of classes enrolled in was only correlated with total number of distractors (*r* = .21, *p* = .028), mind-wandering about future social engagements (*r* = .30, *p* = .001), and somewhat with GPA (*r* = .21, *p* = .079). Controlling for number of classes enrolled in did not change any of the reported results.
